# Predictors of Memory and Processing Speed Dysfunctions after Traumatic Brain Injury

**DOI:** 10.1155/2014/129796

**Published:** 2014-04-29

**Authors:** William Winardi, Aij-Lie Kwan, Tse-Lun Wang, Yu-Feng Su, Chun-Po Yen, Hung-Pei Tsai, Jason Sheehan, Chwen-Yng Su

**Affiliations:** ^1^School of Medicine, Poznan University, 61-701 Poznan, Poland; ^2^Department of Neurosurgery, University of Virginia Health System, Charlottesville, VA 22908, USA; ^3^Department of Surgery, Faculty of Medicine, College of Medicine, Kaohsiung Medical University, Kaohsiung City 807, Taiwan; ^4^Department of Neurosurgery, Kaohsiung Medical University Hospital, Kaohsiung City 807, Taiwan; ^5^Department of Neurosurgery, University of Virginia, Charlottesville, VA 22908, USA; ^6^College of Medicine, Kaohsiung Medical University Hospital, Kaohsiung City 807, Taiwan; ^7^Department of Neurosurgery, University of Virginia Health System, Charlottesville, VA 22908, USA; ^8^Department of Occupational Therapy, College of Health Sciences, Kaohsiung Medical University, Kaohsiung City 807, Taiwan

## Abstract

*Background*. The aims of this study were to evaluate the predictive value of admission Glasgow Coma Scale (GCS) scores, duration of unconsciousness, neurosurgical intervention, and countercoup lesion on the impairment of memory and processing speed functions six months after a traumatic brain injury (TBI) based on a structural equation modeling. *Methods*. Thirty TBI patients recruited from Neurosurgical Department at the Kaohsiung Medical University Hospital were administered the Wechsler Memory Scale-III (WMS-III) and the Wechsler Adult Intelligence Scale-III processing speed index to evaluate the memory and processing speed functions. *Results*. The study showed that GCS scores accounted for 40% of the variance in memory/processing speed. No significant predictive effects were found for the other three variables. GCS classification at the time of TBI seems to correspond moderately to the severity of memory/processing speed dysfunctions. *Conclusions*. The present study demonstrated that admission GCS score is a robust predictor of memory/processing speed dysfunctions after TBI. The results should be replicated with a large sample of patients with TBI, or be extended by examining other potential clinical predictors.

## 1. Introduction

Traumatic brain injury (TBI) which often occurs in adolescents and young adults remains a major issue for public health. The physical and cognitive deficits following TBI often disrupt important developmental processes [1] and psychosocial problems [2, 3]. Therefore, identification of predictors of cognitive recovery from TBI at the acute stage is important in setting realistic expectations of patients' recovery as well as mobilizing appropriate medical and community resources to address patients' needs.

The most commonly reported cognitive dysfunctions in patients with TBI are disturbances in memory and processing speed functions that can persist for years after injury [3–5]. Several premorbid and injury severity factors have been identified to pose a substantial impact on the cognitive sequelae of head injury including age, educational level, presence or absence of neurosurgical intervention, and Glasgow Coma Scale (GCS) scores upon admission [6–9]. Previous studies are toward employing multivariate techniques to predict outcome. The multivariate approaches employed have included variations of multiple regressions, in some cases focusing on a few variables and in others assessing a broader range of predictors. However, conventional multiple regression analysis fails to take measurement errors associated with psychological constructs (i.e., cognition) into account, which can result in estimates of effects that are highly biased due to the influence of error. In addition, multiple regression tests a predictive model with only one dependent variable (i.e., a single test score) which generally does not provide adequate representation of constructs of interest because of imperfect reliability and validity [10].

The structural equation modeling (SEM) is a technique used to specify and estimate models of linear relationships among measured and latent variables [11]. SEM is a superior approach to multiple linear regression analysis as it examines the constructs at the latent level, which provides a more accurate account of the relationships because the relations between theoretically error-free constructs rather than error-prone observed composite variables are estimated [12]. The present study was performed to validate several clinical variables as predictors of memory/processing speed functioning in patients with TBI, using structural equation modeling (SEM).

## 2. Materials and Methods

### 2.1. Participants

A total of 30 patients (24 males, 6 females) with mean age of 32.8 years (range: 16–65 years) with TBI were recruited from neurosurgical outpatient clinic at the Kaohsiung Medical University Hospital in this study. Patients were eligible for the study if they were aged between 16 and 65 years to allow applicability of all available norms of the Chinese versions of the Wechsler Adult Intelligence Scale-Third Edition (WAIS-III) [13] and the Wechsler Memory Scale-Third Edition (WMS-III) [14], were 6 months postonset, and had a Mini-Mental State Examination (MMSE) [15] score > 23 and a Glasgow Coma Scale score of 15 at study inclusion. Patients with multitrauma (e.g., extremity fracture, thoracic injury, etc.), evidence of a prior history of focal brain diseases (e.g., stroke, tumor), serious acute medical illness (heart or renal failure), significant motor impairment, or previous history of dementia, psychiatric disease, Parkinson's disease, or drug and alcohol abuse were excluded. Participants' head injury severity was categorized as “mild,” “moderate,” or “severe” based on the GCS scores at the time of injury. Mild TBI was defined as a loss of consciousness for no greater than 30 minutes and an initial GCS score of 14 to 15, moderate TBI as a GCS score of 9–13, and severe TBI as a GCS score of 3–8 after resuscitation [16]. The demographic and clinical characteristics of the participants are summarized in Table 1.

### 2.2. Neuropsychological Assessments

The processing speed index of the Chinese version of the Wechsler Adult Intelligence Scale-Third Edition (WAIS-III) [13] and the Chinese version of the Wechsler Memory Scale-Third Edition (WMS-III) [14] were used to assess the cognitive impairments after TBI in the domains of processing speed and memory.

The MMSE was used to evaluate general cognitive function in five domains, including orientation to time and place, attention and calculation, registration, short-term recall, and language. The total score ranges from 0 to 30 with a score below 24 indicating cognitive impairment.

### 2.3. Procedure

Demographic, past history, and injury related data were collected via patient interview and examination of the hospital record. All imaging studies were interpreted by a neurosurgeon (ALK) blinded to the findings of the cognitive examination.

A well-trained research assistant administered and scored the WAIS-III, WMS-III, and MMSE in accordance with the standardized procedures as outlined in the manuals. This study was approved by the Kaohsiung Medical University institutional review board. Written informed consent was obtained from all participants.

### 2.4. Statistical Analysis

One-sample *z* test was conducted to assess the differences in Wechsler scales among the TBI patients (mild to moderate group and severe group) and standardization samples of the Wechsler scales. Independent variables included in this analysis were the WAIS-III processing speed index and WMS-III visual immediate and delayed, auditory immediate and delayed, auditory recognition delayed, and working memory indices.

Analysis of Moment Structures (AMOS) software, version 5.0 [17], was used to determine the independent clinical factors associated with memory/processing speed functions. Because the sample size of our study was relatively small, we employed several alternative measures of global fit—the comparative fit index (CFI), nonnormed fit index (NNFI), and root mean square error of approximation (RMSEA). The cut-off values used to assess the adequacy of model fit were determined according to the criteria of MacCallum and Austin [10]. Nonsignificant paths were trimmed from a model described with a series of multiple regression analyses. The fit of the respecified model was tested before being provisionally accepted.

## 3. Results

### 3.1. Patient's Performances on the WAIS-III and WMS-III

Four of the nine WAIS-III and WMS-III index scores fell below the normative mean. Of these, visual immediate index had the lowest mean scores. The indexes below the mean were auditory immediate and delayed, auditory recognition delayed, general memory, and working memory. Overall, our result revealed significant impairment in all of the indices, with the processing speed, visual immediate and immediate memory indexes being the most impaired (Table 2).

Multivariate analysis of variance (MANOVA) was performed to test the severity of TBI injury associated with the WAIS-III and WMS-III performances, and the severity of the injury was significantly associated with WAIS-III and WMS-III performances (*P* < 0.01). To clarify the influence on WAIS-III and WMS-III performances, ANOVAs analyses were performed for seven significant factors.

**Figure 1:**
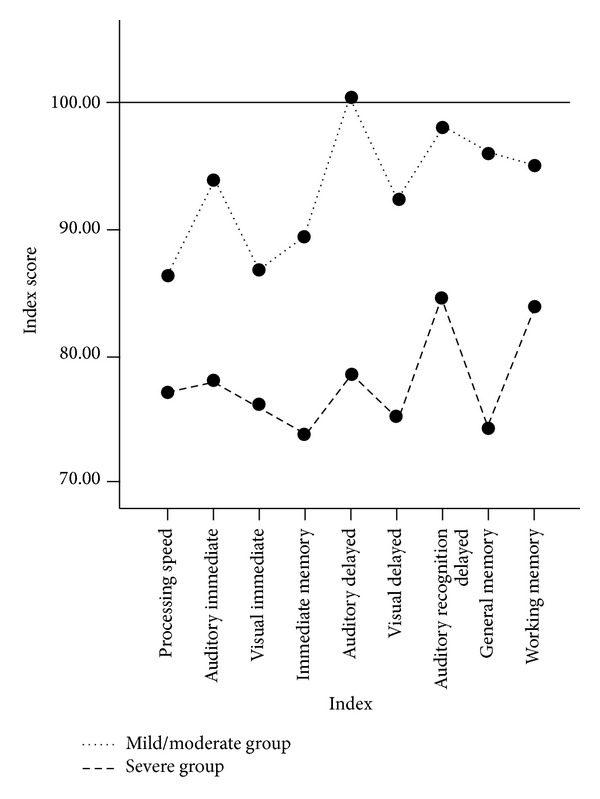
Distribution of index scores for groups with mild to moderate and severe traumatic brain injury.

After the ANOVA analysis, it was determined that patients with mild to moderate TBI group scored significantly higher than severe TBI group on auditory immediate (*P* = 0.001) and auditory delayed (*P* < 0.001) indices.  Figure 1 illustrates the distribution of index scores for the two TBI groups. The discriminant analysis yielded one canonical discriminant function (Wilks' lambda = 0.44; *χ* ^2^ = 20.07; df = 7;* P* < 0.01), accounting for 100% of the discriminating variance. Four variables contributed to the classification of a patient as having mild to moderate TBI, with a standardized discriminant coefficient >0.5, visual immediate (−1.68), visual delayed (1.27), auditory immediate (0.67), and auditory delayed (0.59). The classification results showed that 80% of the original group cases were correctly classified. Group membership was correctly predicted for 78.6% of the patients with severe TBI and 81.3% of the patients with mild to moderate TBI.

### 3.2. Influence of Brain Injury Severity on Memory and Processing Speed

The measurement component of our model was first created, in which the latent variable of memory/processing speed functions was specified by the manifest variables processing speed/working memory, auditory memory, and visual memory. The processing speed/working memory variable was made up of the sum of age-corrected scaled scores for four subtests (letter-number sequencing, spatial span, digit symbol-coding, and symbol search). Working memory is one of the skill components demanded in processing speed. Auditory memory variable was composed of the sum of scaled scores for the immediate and delayed trials of 2 auditory subtests (logical memory and verbal paired associates) as well as the scaled score of the auditory recognition delayed total score. Visual memory variable was composed of the sum of scaled scores for the immediate and delayed trials of 2 visual subtests (faces and family pictures).

Standard goodness-of-fit statistical criteria indicated an excellent fit of the measures to their intended construct (*χ* ^2^ = 1.81,* P* = 0.40, CFI = 1.00, NNFI = 1.01, RMSEA = 0.00). The standardized regression coefficients, which are used to compare the relative importance of the independent variables, for the auditory memory, visual memory, and processing speed/working memory were 0.93, 0.72 and 0.60, respectively. Collectively, these results suggested that our memory/processing speed model was adequately operationalized by successfully identifying variables and latent factor that were clearly related.

SEM regression analysis was performed to test the independent clinical factors (countercoup lesion, three GCS groups, length of coma >7 days versus ≤7 days, and neurosurgical intervention) associated with memory/processing speed latent construct. Significant association was demonstrated for the four independent clinical factors and memory/processing speed latent construct (Table 3). However, using the regression component of our model (Model 1), poor association was demonstrated for the four independent clinical factors and the variance in memory/processing speed (*χ* ^2^ = 16.97,* P* = 0.20). These four predictors together accounted for 44% of the variance in memory/processing speed (*R* ^2^ = 0.44).

**Figure 2:**
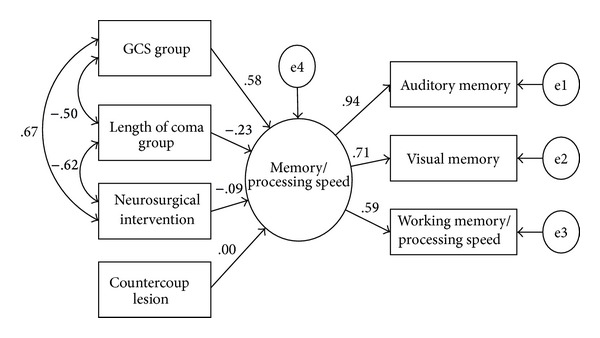
The main structural equation model tested, along with standardized parameter estimates. The rectangles denote observed, endogenous variables or indicators (severity variables and WAIS-III and WMS-III indices), while ellipses to the right of indicators represent measurement errors. The ellipse to the left of the observed variable designates exogenous, latent factor. The values next to the longer single-headed arrows are standardized factor loadings, and the values next to the curved double-headed arrows are correlation coefficients.

To clarify the influence of four independent clinical factors on memory/processing speed and eliminate the confounding effect from other clinical variables, individual regression coefficients analyses were performed for the four significant factors. After the analyses, only GCS group was significantly associated with memory/processing speed (Table 4). A graphic display of this regression model is depicted in Figure 2. As shown in the figure, correlations among GCS group, length of coma group, and neurosurgical intervention were moderate, ranging from −0.50 to 0.67. After removing three nonsignificant paths, global fit of the respective model (Model 2) (CFI = 1.00, NNFI = 1.01, RMSEA = 0.00) markedly improved over Model 1. The squared multiple correlation of memory/processing speed was 0.40, suggesting that GCS grouping explained 40% of memory/processing speed's variance.

## 4. Discussion

To the best of our knowledge, predicting memory outcome using a comprehensive measure of various aspects of memory has never been published. Moreover, our study is not only examining predictive model for different memory functions one at a time but also is enabling data analysis and interpretation in a holistic fashion. GCS reflects the integrity of neuronal function of the brain stem and both cerebral cortices and is widely used as a classification measure of the severity of brain injury [18]. Previous studies [7, 19] showed that the GCS score was significant and independent factors for predicting memory/processing speed dysfunction; our multivariate analysis of the patient data also determined that only GCS grading was a significant factor. Although the impact of GCS was implied in this study, 21% of patients with severe TBI were mistakenly classified into mild to moderate TBI category, whereas 13% of mild to moderate TBI patients were incorrectly classified into severe TBI category. In other words, only 44% of the variance in memory/processing speed construct was explained by 4 TBI severity variables. Thus, our results indicate that factors other than the severity of coma scale may contribute to the low correspondence to the severity of memory/processing speed impairments. Difficult distinguishing of posttraumatic amnesia and coma induced by sedation, small sample size, and suboptimal pooling of the patients with mild and moderate TBI may contribute to this phenomenon, but further study is necessary to determine this.

Traumatic brain injury patients frequently report neurological and psychological symptoms following acute traumatic brain injury. The etiology of these symptoms remains unknown, partly because the symptoms are not specific to TBI, being found in the other clinical conditions and normal individuals. Even though good screening programs are not available of such disease, adequate widespread information could lower the GCS at diagnosis. Length of coma is a commonly reported risk factor for neuropsychological outcome [10, 15, 20]. However, in the present study it was not found to be a significant risk factor. A reason for these discrepancies may be the narrow range of length of coma in our TBI sample, ranging from 0 to 15 days with the exception of one case whose length of coma was 42 days. Another possible reason is that the trauma patterns of these patients are less infiltrative and initially involve the superficial brain tissues, causing conscious disturbance; thus, no neuropsychological dysfunction would be evident on presentation.

Surgical intervention is considered by many investigators to be the best treatment of choice for TBI. Recent retrospective results show that modern anti-increase intracranial pressure agents, combined with good drainage, appear to be associated with a better outcome, including neuropsychological dysfunctions. However, our result demonstrated that surgical intervention had no positive impact on memory/processing speed function in patients with brain injury. Short follow-up time of these patients, associated with the limited number of patients, could explain this observation.

To date, few studies have been published concerning the relationship between presence of a countercoup lesion and memory/processing speed impairments. Ommaya [21] demonstrated that countercoup was associated with the memory/processing speed impairments and served as an unfavorable prognostic factor. However, it remains controversial whether countercoup brain injury could be a significant risk factor for memory/processing speed impairments. In this study, the proportion of patients with countercoup was comparatively low; therefore, it would be absolutely necessary to include a larger number of patients who were accurately diagnosed by CT scan and then followed for a longer period.

To our knowledge, few investigators have analyzed the issues about the differences in visual versus verbal memory impairments as a result of head trauma, and their conclusions remain controversial; some studies reported that visual memory (immediate and delayed) indices were the most impaired in TBI patients [22, 23], whereas others that cited auditory memory indices were the most impaired in TBI sample. For example, Axelrod et al. [24] concluded that WMS-III visual indexes display greater sensitivity to brain dysfunction than the auditory indexes. Because of the small sample size of this study, it is not clear whether the hemispheric laterality has a more detrimental effect on modality-specific (visual versus verbal) memory dysfunctions than GCS group, or vice versa.

In an earlier work by Hoskison et al. [25] prefrontal injury was associated with memory and processing speed dysfunction. In this important study, the authors utilized a preclinical model involving cortical impact injury of rats. Unfortunately, in the clinical setting, traumatic brain injury is seldom as uniform or controlled. In the current study, we did include patients with mild, moderate, and severe TBI. Overall, 53.3% of patients in this study exhibited a countercoup injury. As such, the majority of patients had injury involving two different lobes. In addition, intervening diffuse axonal injury would likely have been observed on MRI sequences designed to evaluate such injury. We believe the current patient population represents a clinically more realistic albeit heterogeneous TBI population. The current study's relationship between GCS and memory/processing speed outcome validates the findings of prior preclinical studies but does so in a clinically representative TBI patient population. Future studies could focus on a more homogenous TBI patient population for evaluation. Also, further studies with larger numbers of TBI patients who with either left, right, or bilateral hemispheric lesions are needed to discern the relative influences of these two variables. Another interesting theme to emerge from our study is the discrepancy between immediate and delayed memory scores. Our study demonstrates a more severe impairment of delayed than immediate memory. This result conforms to those of other studies [23, 26] and may imply that TBI patients generally have retrieval deficit.

The countercoup lesion describes the damage that occurs away from the impact area, suggesting that a shock wave traverses the skull [26]. This secondary reaction can often cause more damage than the initial impact, as shearing of internal tissues and blood vessels leads to further bruising, bleeding, and swelling to the brain. Nevertheless, the effect of this severity variable has not been fully explored in relation to cognitive or functional outcome in patients with TBI.

The present study had several limitations. First, a selection bias could have been present because the study population was small. Second, the present study was retrospective study, and the follow-up time for patients still was short. Third, we could not completely differentiate between duration of unconsciousness due to injury compared to sedation induced coma. Taken together, a multi-institutional prospective study with a large number of patients would be required to confirm the present finding. Furthermore, basic biologic research would be needed to explain the contradictory effects of severity of brain injury on the risk and prognosis of neuropsychological dysfunctions.

In conclusion, our study demonstrated that GCS grading correlates significantly to memory/processing speed outcome but moderately corresponds to severity of memory/processing speed impairments. That is, 21% of severe TBI patients scored in the same range as patients with mild to moderate TBI on memory and processing speed tasks. Our predictive model offers a good vantage point from which similar models can be constructed to cater for the specific nature of outcome measures of interest in future studies.

## Figures and Tables

**Table 1:** Demographic and clinical characteristics of study participants.

	Total sample (*n* = 30)	Mild TBI (*n* = 7)	Moderate TBI (*n* = 9)	Severe TBI (*n* = 14)
Age (years)	32.8 ± 14.2	42.4 ± 14.3	29.1 ± 13.7	30.4 ± 13.2
Male [*n* (%)]	24 (80.0)	6 (85.7)	7 (77.8)	11 (78.6)
Education (years)	11.2 ± 2.4	10.7 ± 2.4	11.2 ± 2.7	11.5 ± 2.3
MMSE scores during the study	27.0 ± 3.6	26.3 ± 5.4	27.1 ± 4.1	27.4 ± 2.0
GCS score at admission	9.7 ± 3.4	14.1 ± 0.7	11.0 ± 1.3	6.6 ± 1.5
Time from injury to cognitive testing, mo	17.4 ± 14.2	8.6 ± 2.8	19.5 ± 13.6	20.6 ± 16.6
Duration of unconsciousness				
<8 days	20 (66.7)	7 (100)	7 (77.8)	6 (42.9)
>7 days	13 (33.3)	0 (0)	2 (22.2)	8 (57.1)
Presence of emergent craniotomy [*n* (%)]	17 (56.7)	0 (0)	5 (55.6)	12 (85.7)
Loss of consciousness [*n* (%)]	27 (90.0)	5 (71.4)	9 (100)	13 (92.9)
Side of brain damage [*n* (%)]				
Right brain	15 (50.0)	4 (57.1)	5 (55.6)	6 (42.9)
Left brain	11 (36.7)	1 (14.3)	3 (33.1)	7 (50.0)
Bilateral	4 (13.3)	2 (28.6)	1 (11.1)	1 (7.1)
Types of brain injury [*n* (%)]				
Closed head injury	21 (70.0)	7 (100)	5 (55.6)	9 (64.3)
Open head injury	9 (30.0)	0 (0)	4 (44.4)	5 (35.7)
Hemorrhagic locations				
Intracerebral	3 (10.0)	1 (14.3)	0 (0)	2 (14.3)
Subarachnoid	7 (23.3)	4 (57.1)	3 (33.3)	0 (0)
Epidural	2 (6.7)	0 (0)	0 (0)	2 (14.3)
Subdural	5 (16.7)	0 (0)	1 (11.1)	4 (28.6)
Multiple sites	13 (43.3)	2 (28.6)	5 (55.6)	6 (42.9)
Presence of countercoup lesion [*n* (%)]	16 (53.3)	3 (42.9)	6 (66.7)	7 (50.0)

GCS: Glasgow Coma Scale; MMSE: Mini-Mental State Examination; TBI: traumatic brain injury.

**Table 2:** Mean WAIS-III and WMS-III index scores, standard deviations, *z*-tests, and rates of impairment for entire sample.

Indexes	Means	SD	*z*-test statistic		
			*z*	*P*	Power
WAIS-III					
Processing speed	82.0	17.4	−6.57	0.00	1.00
WMS-III					
Auditory immediate	86.5	14.0	−4.93	0.00	1.00
Visual immediate	81.9	19.3	−6.61	0.00	1.00
Immediate memory	82.2	17.1	−6.49	0.00	1.00
Auditory delayed	90.4	18.1	−3.51	0.00	0.88
Visual delayed	84.3	20.4	−5.72	0.00	1.00
Auditory recognition delayed	91.8	14.8	−2.98	0.00	0.74
General memory	85.9	17.3	−5.14	0.00	1.00
Working memory	89.9	22.0	−3.68	0.00	0.91

WAIS-III: Wechsler Adult Intelligence Scale-Third Edition; WMS-III: Wechsler Memory Scale-Third Edition; SD: standard deviation.

**Table 3:** Intercorrelations among injury severity variables.

	GCS group	Length of coma group	Neurosurgical intervention
GCS group	—		
Length of coma group	−0.50^a^	—	
Neurosurgical intervention	0.67^a^	−0.62^a^	—
Countercoup lesion	0.02	−0.24	−0.01

GCS: Glasgow Coma Scale.

^a^ *P* < 0.01.

**Table 4:** Parameter estimates for regression models describing effects of injury severity variables on memory/processing speed.

Regression models	Parameter estimates				
	Unstandardized	Standardized	Standard error	Critical ratio	*P* value
Model 1					
Memory/processing speed *←* GCS group	93.09	0.50	38.60	2.41	0.02
Memory/processing speed *←* length of coma group	−111.15	−0.35	65.89	−1.69	0.09
Memory/processing speed *←* surgery	−53.34	−0.18	70.05	−0.76	0.45
Memory/processing speed *←* countercoup lesion	35.99	0.12	48.53	0.74	0.46
Model 2					
Memory/processing speed *←* GCS group	13.36	0.63	3.23	4.13	0.00

GCS: Glasgow Coma Scale.
